# Time in target range of systolic blood pressure and clinical outcomes in atrial fibrillation patients: results of the COOL-AF registry

**DOI:** 10.1038/s41598-024-51385-0

**Published:** 2024-01-08

**Authors:** Rungroj Krittayaphong, Ply Chichareon, Chulalak Komoltri, Ahthit Yindeengam, Gregory Y. H. Lip

**Affiliations:** 1 Division of Cardiology, Department of Medicine, Faculty of Medicine Siriraj Hospital, Mahidol University, 2 Wanglang Road, Bangkoknoi, Bangkok, 10700 Thailand; 2 Cardiology Unit, Department of Internal Medicine, Faculty of Medicine, Prince of Songkla University, Songkla, Thailand; 3 Division of Clinical Epidemiology, Department of Research and Development, Faculty of Medicine Siriraj Hospital, Mahidol University, Bangkok, Thailand; 4 Her Majesty Cardiac Center, Siriraj Hospital, Mahidol University, Bangkok, Thailand; 5 Liverpool Centre for Cardiovascular Science, University of Liverpool, Liverpool John Moores University and Liverpool Heart and Chest Hospital, Liverpool, UK; 6 Department of Clinical Medicine, Aalborg University, Aalborg, Denmark

**Keywords:** Cardiology, Arrhythmias, Hypertension

## Abstract

We aimed to investigate the relationship between time in target range of systolic blood pressure (SBP-TTr) and clinical outcomes in patients with atrial fibrillation (AF). We analyzed the results from multicenter AF registry in Thailand. Blood pressure was recorded at baseline and at every 6 monthly follow-up visit. SBP-TTr were calculated using the Rosendaal method, based on a target SBP 120–140 mmHg. The outcomes were death, ischemic stroke/systemic embolism (SSE), major bleeding, and heart failure. A total of 3355 patients were studied (mean age 67.8 years; 41.9% female). Average follow-up time was 32.1 ± 8.3 months. SBP-TTr was classified into 3 groups according to the tertiles. The incidence rates of all-cause death, SSE, major bleeding, and heart failure were 3.90 (3.51–4.34), 1.52 (1.27–1.80), 2.2 (1.90–2.53), and 2.83 (2.49–3.21) per 100 person-years, respectively. Patients in the 3rd tertile of SBP-TTr had lower rates of death, major bleeding and heart failure with adjusted hazard ratios 0.62 (0.48–0.80), p < 0.001, 0.64 (0.44–0.92), p = 0.016, and 0.61 (0.44–0.84), p = 0.003, respectively, compared to 1st SBP-TTr tertile. In conclusion, high SBP-TTr was associated with better clinical outcomes compared to other groups with lower SBP-TTr. This underscores the importance of good blood pressure control in AF patients.

## Introduction

Non-valvular atrial fibrillation (AF) has an increased risk of not only ischemic stroke but also death, major bleeding, and heart failure^1,2^, even despite the increasing use of oral anticoagulation^3^. Hence, the management of AF has moved beyond simply stroke prevention per se, but towards a holistic or integrated care approach to also cover rate or rhythm treatment, and the control of cardiovascular risk factors and comorbid conditions^4^. Adherence to such an approach is associated with improved clinical outcomes in AF patients^5–8^, leading to its recommendation in international guidelines^9–11^.

Hypertension is a common comorbid condition in patients with AF, with 60–70% of AF patients having hypertension^12,13^. Management of hypertension in AF patients has been associated with improved clinical outcomes^14–16^. Observational cohorts have reported that optimal SBP target in patients with AF was 120–130 mmHg, being associated with the lowest risk of various cardiovascular outcomes^14,17^. In our previous analysis from the COOL-AF registry, we reported that the appropriate optimal SBP target was 120–140 mmHg^16^.

Hence, blood pressure control is crucial to minimize the risk of adverse clinical outcomes in patients with AF, ideally achieving target blood pressure levels as much as possible. We therefore aimed to investigate the relationship between time in target range of systolic blood pressure (SBP-TTr) and clinical outcomes in patients with AF.

## Results

**Figure 1:**
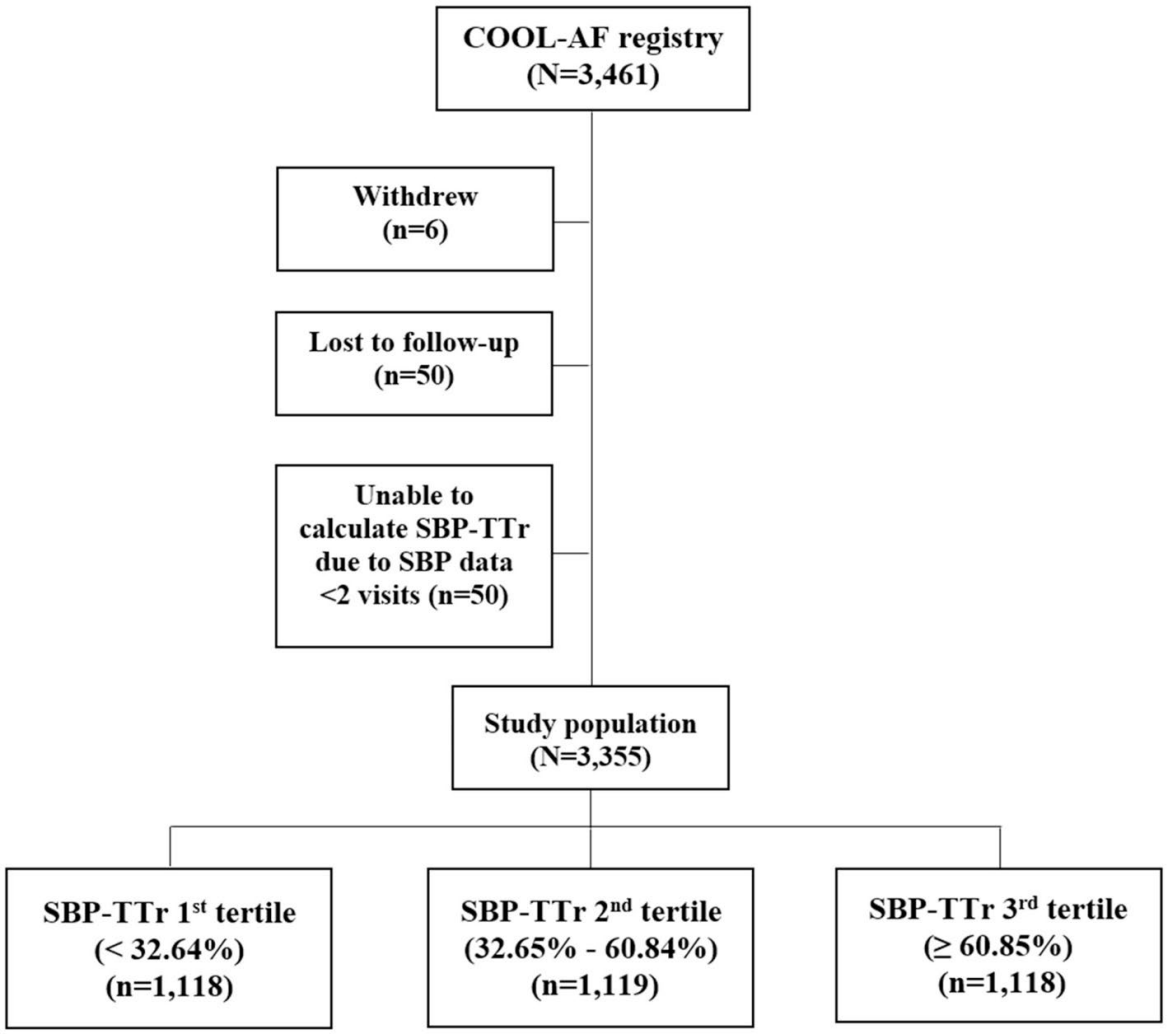
Flow diagram of study population.

A total of 3355 patients were studied, with mean age 67.8 ± 11.2 years, and 1,406 (41.9%) were female. Baseline characteristics of the study population are shown in Table 1. Flow diagram of the study population is shown in Fig. 1. SBP-TTr was classified into 3 subgroups according to the tertiles, as follows: 1st SBP-TTr tertile < 32.84% (n = 1118), 2nd SBP-TTr tertile 32.85–60.84% (n = 1119), and 3rd SBP-TTr tertile ≥ 60.85% (n = 1118). Patients in the highest (i.e. 3rd) SBP-TTr tertile had a lower mean age, lower proportion of females, less cardiovascular risk factors and comorbidities, and lower use of antiplatelets, as well as higher mean BMI, diastolic blood pressure (DBP), CHA_2_DS_2-_VASc and HAS-BLED scores, when compared to other SBP-TTr subgroups.

**Table 1:** Baseline characteristics of study population according to tertiles of time in target range of systolic blood pressure (SBP-TTr).

Variables	All (n = 3,355)	SBP-TTr 1st tertile (n = 1,118)	SBP-TTr 2nd tertile (n = 1,119)	SBP-TTr 3rd tertile (n = 1,118)	p-value
Age (years)	67.8 ± 11.2	67.7 ± 11.9	68.9 ± 11.0	66.7 ± 10.7	**< 0.001**
Female sex	1,406 (41.9%)	500 (44.7%)	468 (41.8%)	438 (39.2%)	**0.029**
Time after diagnosis of AF	3.4 ± 4.3	3.3 ± 4.1	3.4 ± 4.3	3.5 ± 4.6	0.611
Atrial fibrillation					0.082
Paroxysmal	1,133 (33.8%)	366 (32.7%)	381 (34.0%)	386 (34.5%)	
Persistent	634 (18.9%)	239 (21.4%)	209 (18.4%)	186 (16.6%)	
Permanent	1,588 (47.3%)	513 (45.9%)	529 (47.3%)	546 (48.8%)	
Symptomatic AF	2,585 (77.0%)	868 (77.6%)	857 (76.6%)	860 (76.9%)	0.833
History of heart failure	894 (26.6%)	351 (31.4%)	296 (26.5%)	247 (22.1%)	**< 0.001**
History of CAD	541 (16.1%)	201 (18.0%)	183 (16.4%)	157 (14.0%)	**0.039**
CIED	336 (10.0%)	120 (10.7%)	123 (11.0%)	93 (8.3%)	0.067
History of IS/TIA	584 (17.4%)	198 (17.7%)	207 (18.5%)	179 (16.0%)	0.284
Diabetes	830 (24.7%)	257 (23.0%)	307 (27.4%)	266 (23.8%)	**0.034**
Hypertension	2,302 (68.6%)	723 (64.7%)	810 (72.4%)	769 (68.8%)	**< 0.001**
Smoking	688 (19.9%)	231 (20.7%)	208 (18.6%)	229 (20.5%)	0.396
Dyslipidemia	1,890 (56.3%)	600 (53.7%)	644 (57.6%)	646 (57.8%)	0.088
Renal replacement therapy	39 (1.2%)	16 (1.4%)	16 (1.4%)	7 (0.6%)	0.123
Dementia	29 (0.9%)	9 (0.8%)	12 (1.1%)	8 (0.7%)	0.638
History of bleeding	320 (9.5%)	110 (9.8%)	112 (10.0%)	98 (8.8%)	0.555
CHA_2_DS_2_-VASc score					**< 0.001**
Low	285 (8.5%)	93 (8.3%)	71 (6.3%)	121 (10.8%)	
Intermediate	541 (16.1%)	190 (17.0%)	161 (14.4%)	190 (17.0%)	
High	2,529 (75.4%)	835 (74.7%)	887 (79.3%)	807 (72.2%)	
HAS-BLED score					**< 0.001**
0	484 (14.4%)	151 (13.5%)	138 (12.3%)	195 (17.4%)	
1–2	2,339 (69.7%)	757 (67.7%)	803 (71.8%)	779 (69.7%)	
≥ 3	532 (15.9%)	210 (18.8%)	178 (15.9%)	144 (12.9%)	
Antiplatelet	877 (26.1%)	325 (29.1%)	297 (26.5%)	255 (22.8%)	**0.003**
Anticoagulant	2,534 (75.5%)	827 (74.0%)	862 (77.0%)	845 (75.6%)	0.242
Warfarin	2,309 (68.8%)	761 (68.1%)	783 (70.0%)	765 (68.4%)	0.586
NOACs	225 (6.7%)	66 (5.9%)	79 (7.1%)	80 (7.2%)	0.420
Beta blocker	2,443 (72.8%)	818 (73.2%)	838 (74.9%)	787 (70.4%)	0.055
CCB	923 (27.5%)	264 (23.6%)	321 (28.7%)	338 (30.2%)	**0.001**
Digitalis	532 (15.9%)	182 (16.3%)	166 (14.8%)	184 (16.5%)	0.515
MRA	272 (8.1%)	132 (11.8%)	88 (7.9%)	52 (4.7%)	**< 0.001**
Statin	1,986 (59.2%)	646 (57.8%)	663 (59.2%)	677 (60.6%)	0.410
ACEI/ARB	1,533 (45.7%)	528 (47.2%)	534 (47.7%)	471 (42.1%)	**0.013**

Data presented as mean ± standard deviation or number and percentage.

A *p*-value < 0.05 indicates statistical significance.

*SBP-TTr* time in target range of systolic blood pressure, *AF* non-valvular atrial fibrillation, *CIED* cardiac implantable electronic device, *CAD* coronary artery disease, *IS* ischemic stroke, *TIA* transient ischemic attack, *NOACs* Non-vitamin K antagonist oral anticoagulants, *CCB* calcium channel blocker, *MRA* mineralocorticoid receptor antagonists, *ACEI/ARB* angiotensin-converting enzyme inhibitors/angiotensin II receptor antagonists.

Significant values are in bold.

### Clinical outcomes

Average follow-up time was 32.1 ± 8.3 months. The incidence rates of all-cause death, SSE, major bleeding, and heart failure were 3.90 (3.51–4.34), 1.52 (1.27–1.80), 2.2 (1.90–2.53), and 2.83 (2.49–3.21) per 100 person-years, respectively (Table 2).

**Table 2:** Incidence rates of clinical outcomes according to tertiles of time in target range of systolic blood pressure (SBP-TTr) and absolute 3-year risk with death as competing risk.

SBP groups	Number of patients	Number of events	100 person-years	Rate per 100 person-years	Absolute 3-year risk (95% CI) (death as competing risk)
All-cause death	3355	350	89.64	3.90 (3.51–4.34)	
SBP-TTr 1st tertile	1118	155	28.86	5.37 (4.56–6.29)	
SBP-TTr 2nd tertile	1119	104	30.77	3.38 (2.76–4.10)	
SBP-TTr 3rd tertile	1118	91	30.01	3.03 (2.44–3.72)	
SSE	3355	134	88.37	1.52 (1.27–1.80)	4.18 (3.52–4.92)
SBP-TTr 1st tertile	1118	54	28.28	1.91 (1.43–2.49)	5.08 (3.86–6.54)
SBP-TTr 2nd tertile	1119	38	30.49	1.25 (0.88–1.71)	3.55 (2.56–4.79)
SBP-TTr 3rd tertile	1118	42	29.60	1.42 (1.02–1.92)	3.92 (2.86–5.22)
Major bleeding	3355	193	87.73	2.20 (1.90–2.53)	6.01 (5.22–6.88)
SBP-TTr 1st tertile	1118	73	28.19	2.59 (2.03–3.26)	6.95 (5.50–8.61)
SBP-TTr 2nd tertile	1119	71	29.97	2.37 (1.85–2.99)	6.38 (5.02–7.94)
SBP-TTr 3rd tertile	1118	49	29.58	1.66 (1.23–2.19)	4.71 (3.54–6.13)
Heart failure	3355	245	86.46	2.83 (2.49–3.21)	7.68 (6.79–8.65)
SBP-TTr 1st tertile	1118	109	27.44	3.97 (3.26–4.79)	10.43 (8.67–12.38)
SBP-TTr 2nd tertile	1119	78	29.69	2.63 (2.08–3.28)	7.23 (5.78–8.88)
SBP-TTr 3rd tertile	1118	58	29.33	1.98 (1.50–2.56)	5.45 (4.18–6.95)

*SBP-TTr* time in target range of systolic blood pressure, *SBP* systolic blood pressure, *SSE* ischemic stroke/systemic embolism, *CI* confidence interval.

**Figure 2:**
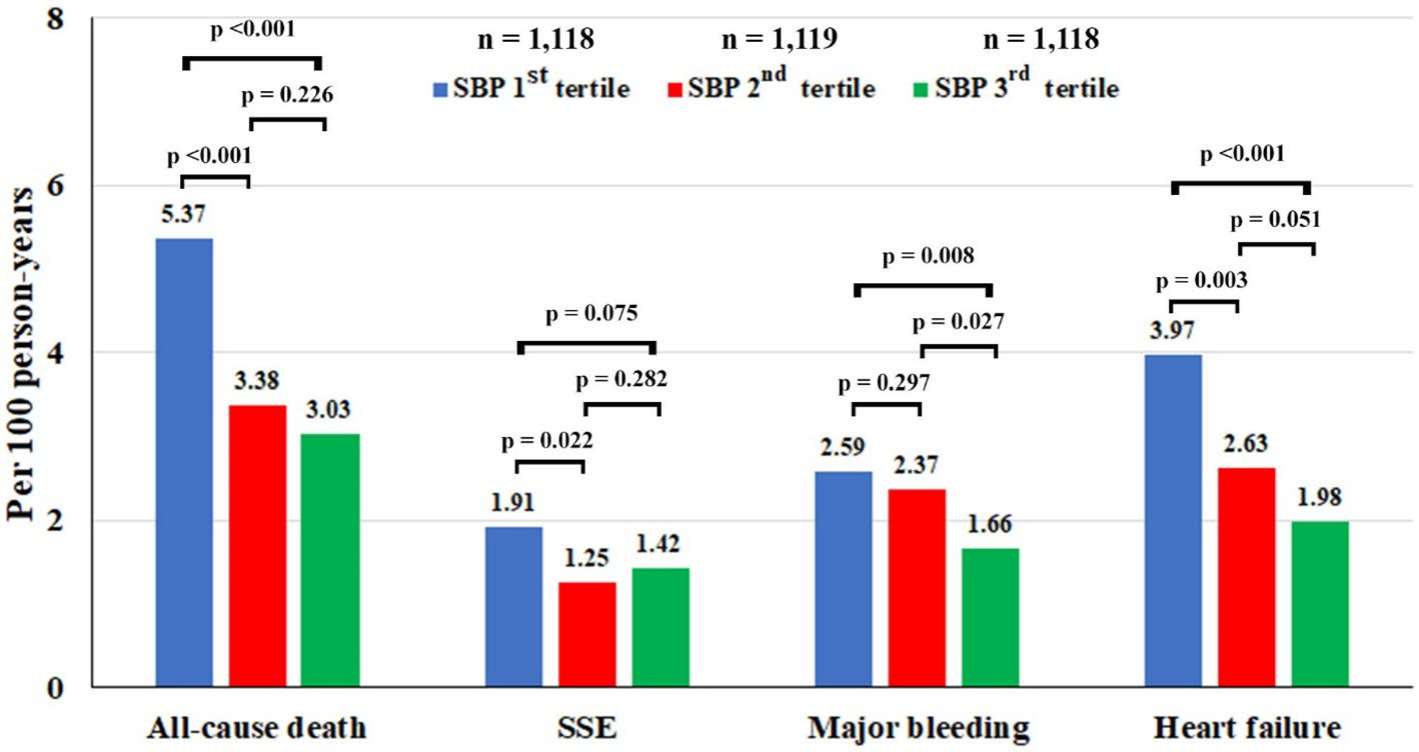
Incidence rate of all-cause death, ischemic stroke/systemic embolism (SSE), major bleeding, and heart failure according to tertiles of time in target range of systolic blood pressure (SBP-TTr).

**Figure 3:**
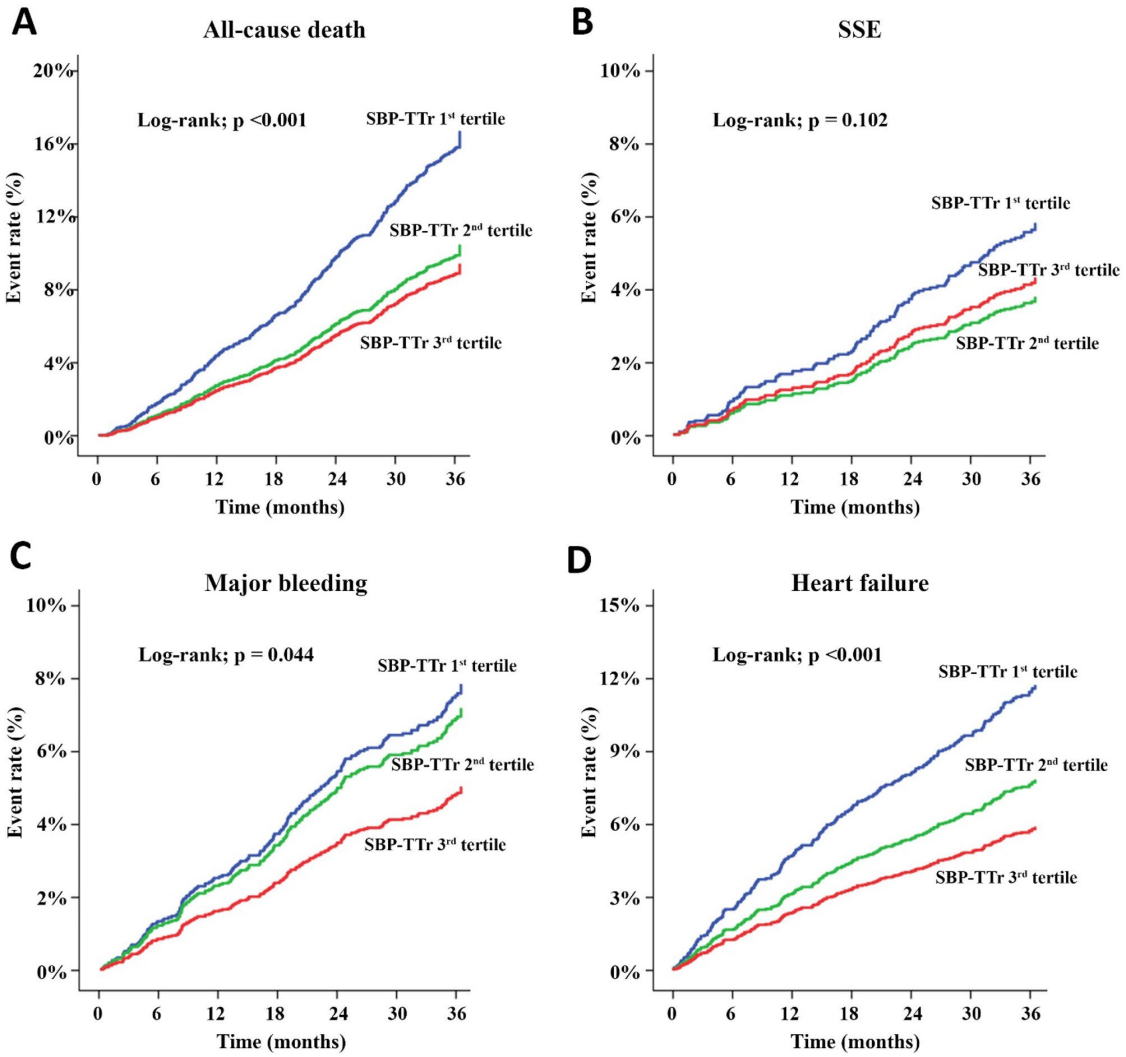
Cumulative event rate over time for all-cause death (**A**), ischemic stroke/systemic embolism (SSE) (**B**), major bleeding (**C**), and heart failure (**D**) according to tertiles of time in target range of systolic blood pressure (SBP-TTr).

Patients in the 3rd tertile of SBP-TTr had lower rates of death, major bleeding and heart failure and tended to have a lower rate of SSE when compared to the other groups (Fig. 2). Figure 3 shows hazard graph of cumulative event rates of all-cause death, SSE, major bleeding, and heart failure. Patients in the 1st SBP-TTr tertile had the highest event rates for all clinical outcomes and the 3rd SBP-TTr tertile had the lowest rates for all-cause death, major bleeding, and heart failure and for SSE, similar to 2nd SBP-TTr tertile and lower than 1st SBP-TTr tertile.

### Multivariable analysis

**Figure 4:**
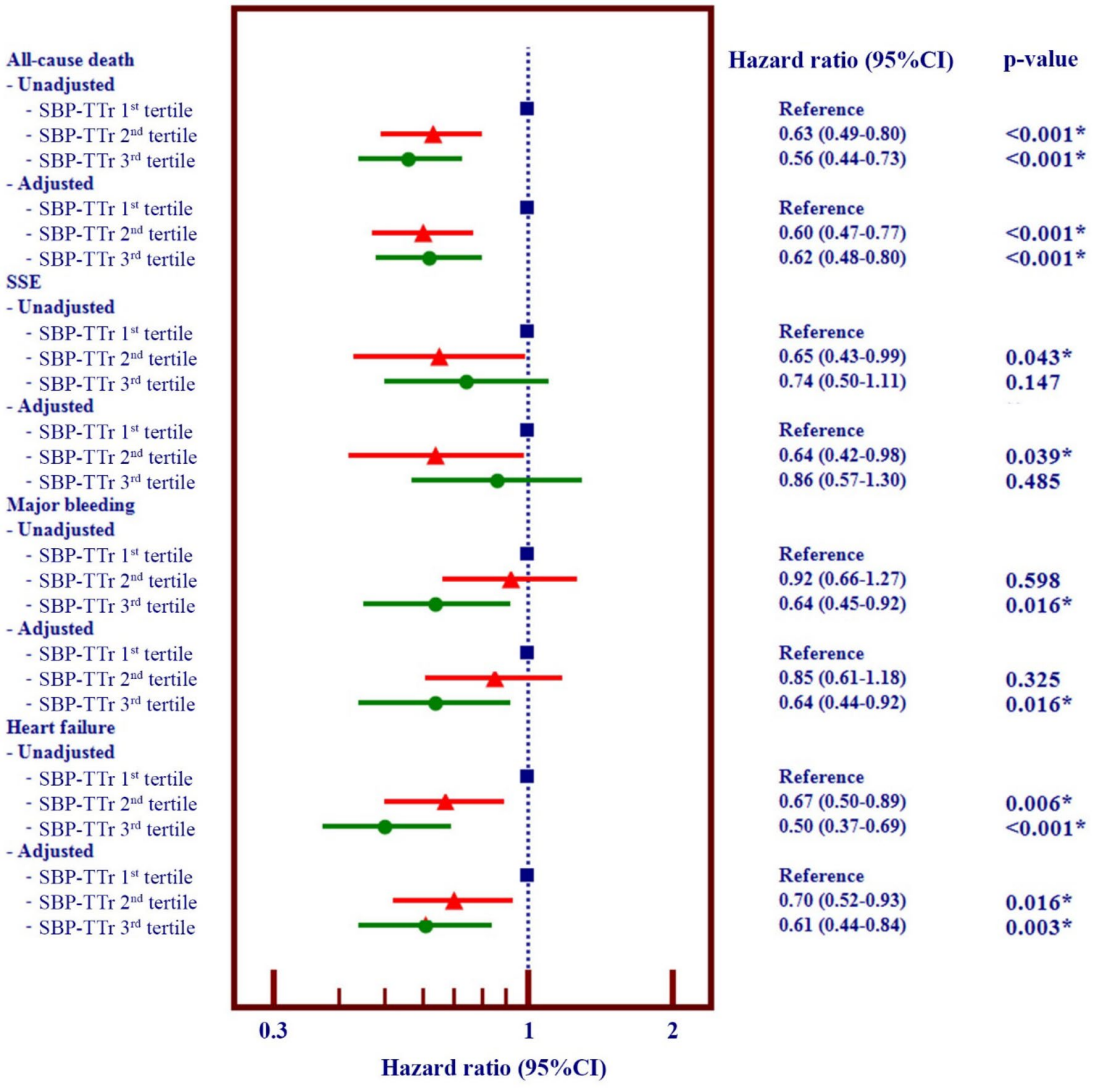
Forest plot of unadjusted and adjusted hazard ratio (HR) and 95% confidence interval (CI) for all-cause death, ischemic stroke/systemic embolism (SSE), major bleeding, and heart failure according to tertiles of time in target range of systolic blood pressure (SBP-TTr).

Multivariable analysis was performed for the assessment of effect of SBP-TTr on each clinical outcome. All baseline variables were used for the adjustment. Unadjusted and adjusted hazard ratios and 95% confidence intervals are shown in Fig. 4. Forest plots demonstrate that patients in the 3rd SBP-TTr tertile had the lower rates for all-cause death, major bleeding, and heart failure, both for unadjusted and adjusted analysis. Patients in the 2nd and 3rd SBP-TTr tertiles tended to have a lower rate of SSE compared to those in the 1st SBP-TTr tertile.

### Sensitivity analysis

Sensitivity analysis was performed by categorizing SBP-TTr into 2 groups: < 65% (n = 2396) and ≥ 65% (n = 959). The results of unadjusted and adjusted Cox proportional hazard model showed that patients with SBP-TTr ≥ 65% had lower rates of all-cause death, major bleeding and heart failure, when compared to those with SBP-TTr < 65% (Supplementary Fig. 1).

**Figure 5:**
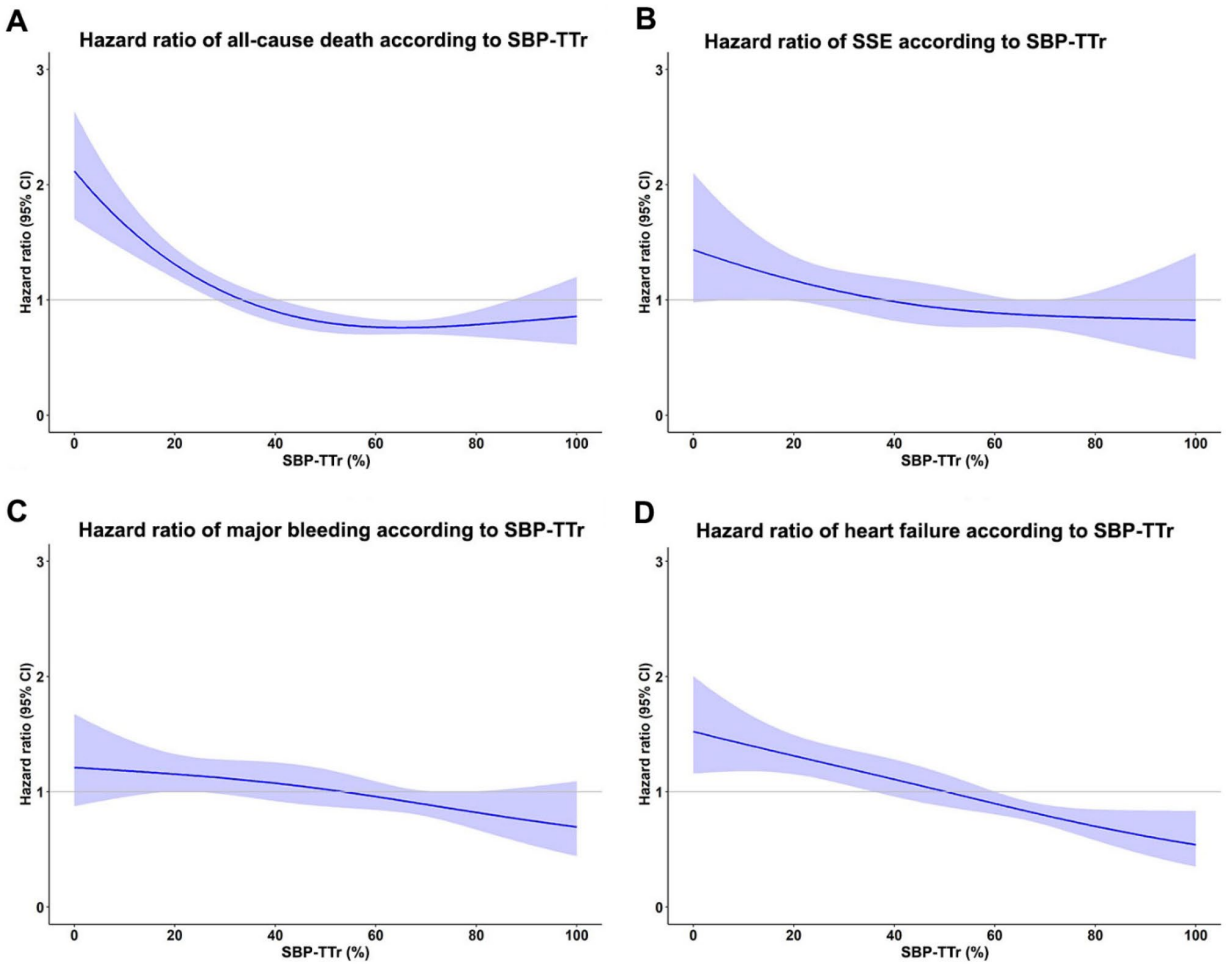
Restricted cubic spline graph of adjusted hazard ratio for (**A**) all cause death, (**B**) ischemic stroke/systemic embolism (SSE), (**C**) major bleeding, and (**D**) heart failure relative to time in target range of systolic blood pressure (SBP-TTr) as continuous data.

Additional analyses were performed by treating SBP-TTr as continuous data, and by using restricted cubic spline graphs to display the relationship between TTR and all-cause death, major bleeding and heart failure (Fig. 5). This shows that higher SBP-TTr was associated with a lower risk of all-cause death, major bleeding and heart failure and tended to be associated with a lower risk of SSE as well.

## Discussion

The principal result of this multicenter prospective nationwide registry of Thai patients with AF demonstrates that AF patients in the 3rd SBP-TTr tertile (SBP target 120–140 mmHg) was associated with the best clinical outcomes (all-cause death, major bleeding, and heart failure), while those in the 1st tertile had the worst outcomes.

The calculation of SBP-TTr was based of the SBP data of every visit. This result underscores the importance of good blood pressure control, and maintaining patients within recommended treatment targets. This is important, since hypertension is a major risk factor which can lead to adverse cardiovascular outcomes such as stroke and adverse cardiovascular events in patients with AF^17^. In general populations, the risk of cardiovascular events was double for every 20 mmHg increase of SBP starting from SBP of 115–175 mmHg^18^. Conversely, a reduction in SBP of 10 mmHg can reduce cardiovascular events by 20%^19^. However, less than 1 in 7 of patients with hypertension are well controlled mainly due to unawareness and therapeutic inertia^20^. Also, too low SBP may increase risk of cardiovascular events especially in patients with CAD^21^ or diabetes^22^, consistent with a J-curve phenomenon. Despite the results from SPRINT study showing that target SBP less than 120 mmHg had a better outcome than 140 mmHg^23^, many guidelines still recommended that optimal SBP should be 120–130 mmHg^24,25^ which indicates that the SBP should be controlled but not be too low. Indeed, previous studies related to optimal SBP in AF patients with hypertension have shown that the target SBP should be 120–130 mmHg^17^ or 120–140 mmHg^16^.

The calculation of SBP-TTr in this study followed the Rosendaal method^26^ that has been used to calculate time in therapeutic range of INR in patients who use warfarin therapy. The results demonstrated that patients in the 3rd tertile of SBP-TTr had a better clinical outcome in terms of all-cause death, major bleeding and heart failure compared to other subgroups, and patients in the 1st SBP-TTr tertile had the worst outcomes. For SSE, the result of SBP-TTr was not statistically significant due to a similar rate of clinical outcomes in patients with 2nd and 3rd tertile; however, patients in the 1st SBP-TTr tertile also had a trend toward the worst SSE outcomes.

The better clinical outcomes in AF patients in the 3rd tertile of SBP-TTr was not only related to all-cause death but also to major bleeding and heart failure. For major bleeding, the explanation may be that AF patients in the 3rd tertile of SBP-TTr had a better control of SBP, thereby lower risk of bleeding which is known to be increased in AF patients with high blood pressure^27^. Similarly, uncontrolled hypertension has been associated with an increased risk of acute decompensated heart failure^28^. Indeed, one study from Korea demonstrated a reverse J-curve of the relation of SBP and heart failure, which was apparent both for reduced and preserved ejection fraction^29^.

### Limitations

This study had some limitations. First, the study sites were mainly university hospitals or large tertiary hospitals, and therefore, the results may not be applied for other settings, such as community based AF patients. Second, despite the SBP in patients with AF having more fluctuations compared to patients in sinus rhythm, previous studies and meta-analysis have shown that SBP measurement in patients with AF remains fairly reliable^30,31^. Third, the number of SBP readings over the study period may be relatively small. Since this is the prospective registry in nature, we believed that the number of SBP records is reasonable and should be able to make analysis to answer the objective of this study. Studies that had SBP data from several time points usually come from retrospective analysis of national data such as insurance data. The benefits of registry type of study such as our study were (1) the data collection were well plan before the data acquisition since we plan to measure blood pressure 3 time and make the average, (2) clinical outcome were prospective collected, more reliable and well adjudicated.

## Conclusion

High SBP-TTr was associated with better clinical outcomes compared to other groups with lower SBP-TTr. This underscores the importance of good blood pressure control in patients with AF, and emphasizes the need for a holistic or integrated care approach to AF management that includes optimization of cardiovascular risk factors and comorbidities.

## Methods

### Study population

This study was an ancillary analysis from The COhort of antithrombotic use and Optimal INR Level in patients with non-valvular atrial fibrillation in Thailand (COOL-AF) registry. This was a prospective nationwide registry of patients with non-valvular AF in 27 hospitals in Thailand. Patients were enrolled during 2014–2017 and the planned follow-up was 3 years. The inclusion criteria were patients who were diagnosed as AF, age at least 18 years, and had an ECG documentation of AF. The exclusion criteria were as follows: (1) rheumatic valvular disease; (2) mechanical heart valve; (3) AF from transient reversible cause; (4) history of ischemic stroke within 3 months; (5) hematologic disease that increased risk of bleeding such as thrombocytopenia or myeloproliferative disease; (6) life expectancy less than 3 years; (7) unable to come for the follow-up visit; (8) current hospitalization; (9) refusal to provide informed consent; (10) participation in a clinical trial; and (11) unable to calculate SBP-TTr. This study was approved by the Central Research Ethic Committee (CREC) with the Certificate of Approval (COA) number CREC 003/2014. The study was conducted in accordance to the principles set forth in the Declaration of Helsinki and the International Conference on Harmonization for Good Clinical Practice Guidelines. All patients provided written informed consent before participation.

### Study protocol

Investigators were instructed to enroll patients consecutively. The required data of the baseline visit were retrieved from the medical record and by patient interview. All data were recorded in the case record form as a hard copy and also typed into a web-based electronic case record system. All data were validated with double-entry method. Investigators were required to collect data during the follow-up visits at 6, 12, 18, 24, and 30 months. Required data were written in the hard copy and typed in the web-system similar to the baseline visit. Data verification and clarification was performed accordingly.

### Data collection

The following data were collected at baseline: (1) age, sex; (2) vital signs; (3) details of AF including symptoms and duration of AF; (4) cardiovascular risk factors such as hypertension, smoking, dyslipidemia, and type 2 diabetes; (5) comorbid conditions such as history of coronary artery disease (CAD), chronic kidney disease (CKD); (6) ECG data; (7) laboratory data; (8) previous investigation such as echocardiographic data; and (9) medication data including oral anticoagulants and antiplatelets. Components of the CHA_2_DS_2-_VASc score [**C** = congestive heart failure (1 point); **H** = hypertension (1 point); **A** = age > 75 years (2 points); **D** = diabetes (1 point); **S** = stroke (2 points); **V** = vascular disease (1 point); **A** = age 65–74 (1 point); and **Sc** = female sex category (1 point)]^32^ and HAS-BLED score [uncontrolled **H**ypertension, **A**bnormal renal, or liver function; history of **S**troke; history of **B**leeding; **L**abile INR; **E**lderly (age above 65 years); and, **D**rugs or alcohol (1 point each)]^33^ were recorded.

Blood pressure was recorded at baseline and at every follow-up visit. Investigators were instructed measure blood pressure according to guideline recommendations^34^. To improve accuracy of blood pressure measurement in patients with AF, investigators were encouraged to take 3 blood pressure measurements and recording the average^31^. Data from the follow-up visits were collected at a similar manner but also included the clinical outcome data.

### Outcomes

The main outcome measurements were death, ischemic stroke/systemic embolism (SSE), major bleeding, and heart failure (HF). Documents relating to clinical outcome were uploaded into the web-based system. Outcome data were confirmed by an adjudication committee.

Ischemic stroke was defined as sudden-onset neurologic deficit lasting greater than 24 h or transient ischemic attack (TIA) for the duration of the neurologic deficit less than 24 h. Whether positive or negative, imaging data from computerized tomography (CT) brain scan or magnetic resonance imaging (MRI) were required to be uploaded into the web-based system. Systemic embolism was defined as a clinical and objective evidence of the sudden loss of end-organ perfusion. Major bleeding was defined using International Society of Thrombosis and Haemostasis (ISTH) criteria^35^. A HF event was defined as a hospital admission or a presentation of the patient for an urgent, unscheduled visit with a primary diagnosis of HF, whereby the patient exhibits new or worsening symptoms of HF on presentation, has objective evidence of new or worsening HF, and received initiation or intensification of treatment specifically for HF^36^.

Investigators were required to upload essential documentation to support diagnosis of the clinical outcomes into the web-based system. All supporting documents in the web system were sent to the adjudication committee to confirm the diagnosis.

### Statistical analysis

Continuous data are described as mean and standard deviation (SD),while categorical data are presented as number and percentage. SBP-TTr was calculated by the Rosendaal method used of the calculation of time in therapeutic range of international normalized ratio (INR)^26^. The periods between 2 consecutive SBP recordings were linearly interpolated and each day was given an SBP value. We collected SBP value and the date of SBP of every SBP data. From the 2 adjacent SBP, we calculated the percentage of time between the 2 SBP that have the SBP within target range of 120–140 mmHg. We then calculated the days within target range of the 2 SBP intervals from the percentage of time within the target range divided by number of days between the 2 SBP data. After that, we made a sum of days within the target range of every interval between the 2 SBP. SBP-TTr derived from the overall days within target range divided by the total number of days from the first SBP to the last SBP. Continuous data among the 3 SBP-TTr subgroups were compared by analysis of variance (ANOVA) test with Bonferroni post hoc analysis. Comparisons of categorical data among the 3 groups were performed by chi-square test with Bonferroni post hoc analysis. Incidence rates of clinical outcomes were described by rate per 100 person-years and 95% confidence interval (CI). The results of survival analysis of clinical outcomes compared among the 3 average SBP groups were compared using log-rank test.

Cox proportional hazards model (enter method) was used to perform multivariable analysis to determine the effect of average SBP on each clinical outcome. Multivariable analysis was performed using age, sex, type of AF, symptomatic AF, history of heart failure, history of CAD, cardiac implantable electronic device (CIED), history of ischemic stroke/TIA, diabetes, hypertension, smoking, dyslipidemia, renal replacement therapy, dementia, history of bleeding, antiplatelet, and OAC as covariates for the adjustment. The results were shown as Hazard ratios and 95% confidence intervals. Cox models were also used to calculate the rate of clinical outcome with ‘death without event’ considered to be a competing risk.

Sensitivity analysis was performed by (1) comparing clinical outcomes of SBP-TTr as 2 groups (< and ≥ 65%), (2) analyzing outcomes in relation to SBP-TTr values as a continuous variable, using cubic spline curves. A two-sided p-value less than 0.05 was considered statistically significant. All analyses were performed using SPSS statistical software version 18.0 (SPSS, Inc., Chicago, IL, USA) and R version 3.6.3 (http://www.r-project.org).

### Supplementary Information

Supplementary Figure 1.

## Data Availability

The dataset that was used to support the conclusion of this study is included within the manuscript. Any other additional data will be made available upon request to the corresponding author.
