# Supplementary material for: Time in target range of systolic blood pressure and clinical outcomes in atrial fibrillation patients: results of the COOL-AF registry
Source: Sci Rep. 2024 Jan 8;14:805. doi: 10.1038/s41598-024-51385-0 (PMC10774389; doi:10.1038/s41598-024-51385-0)

**Supplementary materials**

**Supplementary Figure 1.** Forest plot of unadjusted and adjusted hazard ratio (HR) and 95% confidence interval (CI) for all-cause death (A), ischemic stroke/systemic embolism (SSE)(B), major bleeding (C), and heart failure (D) compared between time in target range of systolic blood pressure (SBP-TTr) ≥65% and <65%.


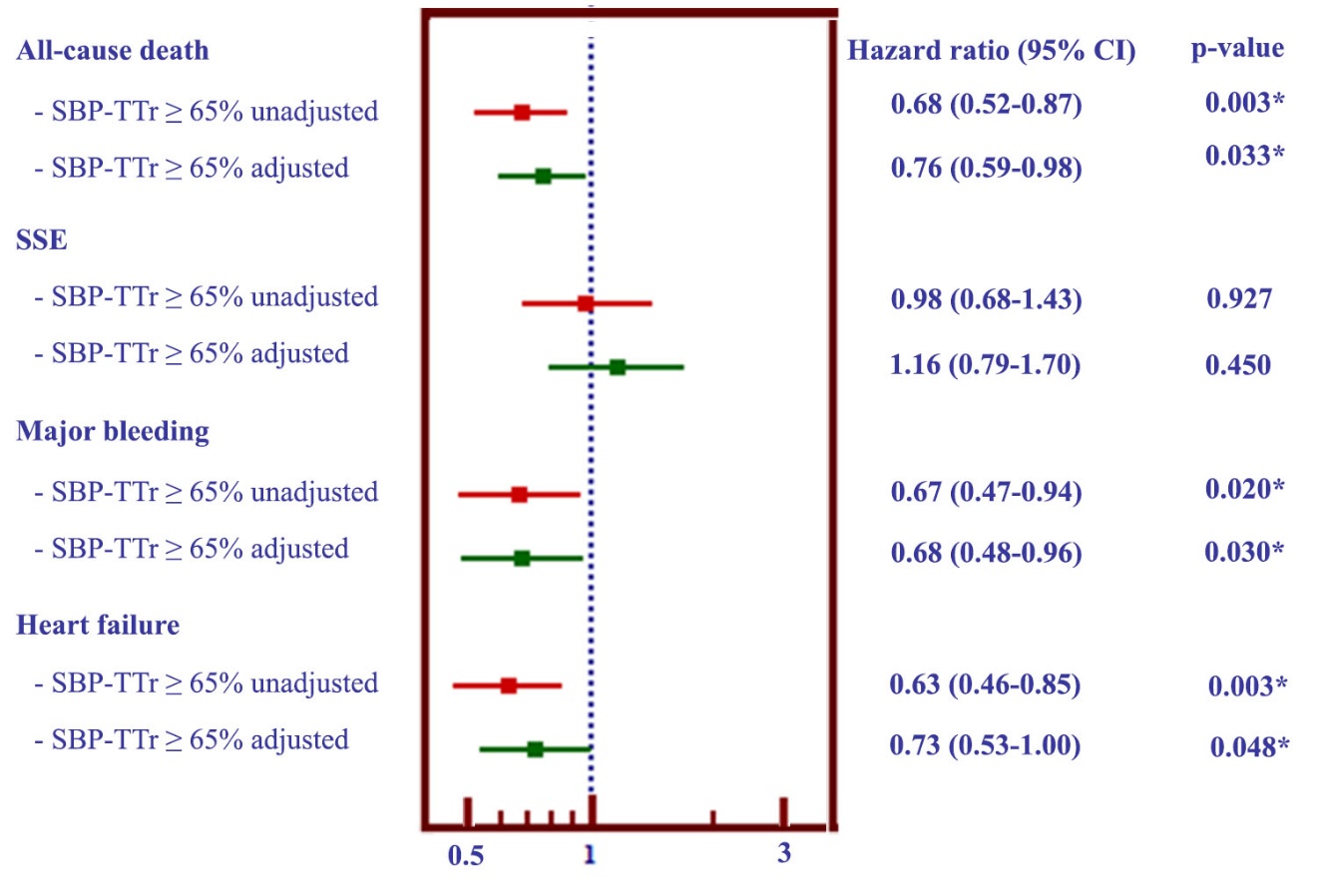

Supplement: Supplementary file 1 — Supplementary Figure 1. [file 41598_2024_51385_MOESM1_ESM.docx]
